# Raising Awareness on the Clinical and Forensic Aspects of Jellyfish Stings: A Worldwide Increasing Threat

**DOI:** 10.3390/ijerph19148430

**Published:** 2022-07-10

**Authors:** Sara Almeida Cunha, Ricardo Jorge Dinis-Oliveira

**Affiliations:** 1 Department of Public Health and Forensic Sciences, and Medical Education, Faculty of Medicine, University of Porto, 4200-319 Porto, Portugal; up201603632@edu.med.up.pt or; 2 TOXRUN—Toxicology Research Unit, University Institute of Health Sciences (IUCS), CESPU, CRL, 4585-116 Gandra, Portugal; 3 UCIBIO-REQUIMTE—Applied Molecular Biosciences Unit, Laboratory of Toxicology, Department of Biological Sciences, Faculty of Pharmacy, University of Porto, 4050-313 Porto, Portugal; 4 MTG Research and Development Lab, 4200-604 Porto, Portugal

**Keywords:** jellyfish, pathophysiology, signs and symptoms, clinical and forensic diagnosis, treatment

## Abstract

Jellyfish are ubiquitous animals registering a high and increasing number of contacts with humans in coastal areas. These encounters result in a multitude of symptoms, ranging from mild erythema to death. This work aims to review the state-of-the-art regarding pathophysiology, diagnosis, treatment, and relevant clinical and forensic aspects of jellyfish stings. There are three major classes of jellyfish, causing various clinical scenarios. Most envenomations result in an erythematous lesion with morphological characteristics that may help identify the class of jellyfish responsible. In rare cases, the sting may result in delayed, persistent, or systemic symptoms. Lethal encounters have been described, but most of those cases happened in the Indo-Pacific region, where cubozoans, the deadliest jellyfish class, can be found. The diagnosis is mostly clinical but can be aided by dermoscopy, skin scrapings/sticky tape, confocal reflectance microscopy, immunological essays, among others. Treatment is currently based on preventing further envenomation, inactivating the venom, and alleviating local and systemic symptoms. However, the strategy used to achieve these effects remains under debate. Only one antivenom is currently used and covers merely one species (*Chironex fleckeri*). Other antivenoms have been produced experimentally but were not tested on human envenomation settings. The increased number of cases, especially due to climate changes, justifies further research in the study of clinical aspects of jellyfish envenoming.

## 1. Introduction

Jellyfish is a designation given to the free-swimming pelagic state of some members of the phylum Cnidaria [1]. Although most species live in tropical and temperate waters, they can also be found in cold marine environments. It is known that jellyfish populations go through major oscillations every 20 years [2], and their numbers have been increasing globally in recent years, with more geographical areas affected and an increased number of outbreaks [3]. The causes of the current population surge are yet to be clarified, however, global warming-induced warmer marine water temperatures, overfishing of natural predators and industrialization seem to play a role [4]. Of the roughly 10,000 jellyfish species, approximately 100 are accountable for the majority of human envenomations. Around 2006, 150 million cnidarian envenomations are estimated to occur [5]. Cnidarian venom is responsible for local as well as systemic symptoms, though the severity varies greatly, depending on the animal, area of the sting, and individual susceptibility [6]. The encounters with jellyfish can range from a mere nuisance to lethal events. As jellyfish populations are rising due to climate changes and traveling becomes increasingly more accessible, it is expected that more clinicians worldwide will encounter patients stung by jellyfish. As such, it is increasingly important that physicians and forensic experts all over the world are familiar with the clinical picture of jellyfish envenomation, its treatment, and possible complications. Previous studies of the research group have been highlighting the relevance of image in the clinical and forensic suspicion and different aspects of toxicology have been reviewed in the last few years [7,8,9,10,11]. This work follows this major objective by fully reviewing the state-of-the-art concerning pathophysiology, diagnosis, treatment, and relevant clinical and forensic features of jellyfish stings.

## 2. Materials and Methods

A thorough search in PubMed (U.S. National Library of Medicine) was undertaken without a date or language constraint, focusing on pathophysiology, signs and symptoms, history and physical examination, diagnostic, treatment, and forensic aspects of jellyfish stings. Furthermore, the keyword “jellyfish” was crossed with syndrome, poisoning, envenomation, scar, and cnidaria. A total of 232 scientific documents were considered for this review offering a complete overview of major clinical and forensic aspects of jellyfish stings.

## 3. Jellyfish Biology

The phylum Cnidaria encompasses six classes: Scyphozoa, Hydrozoa, Cubozoa, Anthozoa, Myxozoa, and Staurozoa [12]. Of these, only Scyphozoa, Hydrozoa and Cubozoa contain animals referred to as jellyfish. **Scyphozoans** are considered “true jellyfish” and encompass most of the jellyfish species. They can be found worldwide, and their stings are usually mild. Some of the Scyphozoan with medical relevance include the *Pelagia noctiluca*, *Aurelia aurita*, *Chrysaora quinquecirrha, Linuche unguiculata, Nemopilema nomurai, Rhizostoma pulmo*, and *Cyanea capillata*. Only a few members of the class **Hydrozoa** are regarded as jellyfish. *Physalia physalis* (also known as Portuguese man o’war or the man-of-war) is probably the most relevant member of the class, as it is responsible for most hydrozoan envenomations and elicits a distinctive clinical picture. It is not a single organism, but a colony that operates as a single individual. *Olindias sambaquiensis* is also a Hydrozoan jellyfish but it is only found in Brazil. **Cubozoa** includes the deadliest jellyfish species. They have cube-shaped bodies and usually inhabit tropical and subtropical oceans. The most dangerous species, namely *Carukia barnesi,* and *Chironex fleckeri* are, however, restricted to the Indo-Pacific region. Jellyfish bell sizes range from about 1 mm to several centimeters (excluding the tentacles and oral arms). *Cyanea capillata* (scyphozoan) is the biggest known jellyfish, with tentacles capable of reaching 36.5 m in length [13]. These animals are equipped with a special type of stinging cell, the nematocyte or cnidocyte, used for defense, capturing prey, and spatial competition. It contains many extrusive organelles derived from the Golgi apparatus, called nematocysts. These consist of a casing with a hinged operculum, inside which an inverted coiled tubule is immersed in the jellyfish venom. Mechanical or chemical stimulation prompts the tubule’s quick eversion, therefore inoculating the venomous substances [14]. The nematocysts are present in several regions of the cnidarian’s body; they are most often seen on tentacles and oral arms, but they can also be found on the bell [6]. These organelles are responsible for human envenomation.

## 4. Toxicokinetics of Jellyfish Venom

Not much is known about the toxicokinetics of this venom. Jellyfish venom enters the human body through the epidermis when it is pierced by a nematocyst tubule. It should be noted that these organelles may remain viable even in dead organisms and in small fragments of tentacles that broke loose from the animal [15]. The venom exerts its effects locally, but it can also enter the bloodstream, causing systemic symptoms.

In Irukandji syndrome (caused by envenomation from Cubozoan jellyfish), the reason for the delay between the sting and the manifestation of the systemic symptoms is suspected to be related to the path the toxins take to reach their target. *Carukia barnesi* venom has large elements (50–100 kDa) and may thus move through the lymphatic system, in a route similar to that of snake venoms, giving the distinctive 30 min delay in the onset of symptoms [16].

## 5. Pathophysiology

Cnidarian venom consists of a complex combination of bioactive elements, including components such as serotonin and histamine, along with high molecular weight proteins. Of these, there have been described lipases, proteases, serine protease inhibitors, hyaluronidases, deoxyribonucleases, l-amino acid oxidases, c-type lectins, neurotoxins, ion channel blockers, pore-forming toxins, and cysteine-rich secretory protein [14].

The effect of the jellyfish venom seems to be predominantly toxic in nature. However, it has been described that some components of the venom can act as antigens, eliciting an innate immune response, antibody formation, and activating the immunological memory [17,18,19,20].

Despite extensive research, the pathophysiological processes and mechanisms of this venom remain unknown. In general, cardiotoxicity is thought to be the leading cause of mortality [21], whereas hemolytic activity seems to be a preliminary damaging factor, providing a method for disentangling the complex venom. Cardiovascular toxicity is, however, independent from hemolysis [22]. Hemolysis is a common effect of several jellyfish venoms. Some venom components attain a hemolytic effect by altering cell permeability, causing ion currents, cell swelling, and osmotic lysis, while others degrade the phospholipids bilayer or form pores in the membrane [23,24,25].

The best-described jellyfish toxic activity is cell lysis by pore-forming toxins [26]. There have also been isolated neurotoxins, targeting ionic channels and neurotransmitter receptors [27]. Oxidative stress has also been reported as a pathophysiologic mechanism [28].

### 5.1. Cardiotoxicity, Pore Formation, and Intracellular Ca^2+^ Overload

Jellyfish venom cardiac effect shows great variation, ranging from no apparent cardiotoxicity to raised troponin I levels [29], Tako-Tsubo cardiomyopathy [30], and acute myocardial infarction [31]. Some species seem to be particularly cardiotoxic, such as *Carukia barnesi*, responsible for the Irukandji syndrome. Patients with Irukandji syndrome, despite having no or minor skin markings, have substantial and continuous pain, tachycardia, hypertension followed by hypotension, and pulmonary edema, implying serious cardiac dysfunction [32,33].

Although the precise mechanism of the acute cardiac malfunction is yet to be fully understood, intracellular Ca^2+^ excess caused by extracellular Ca^2+^ entry through pore-forming toxins, as well as Ca^2+^ intracellular release led by β adrenergic signaling have been identified as key factors driving the cardiotoxicity of jellyfish venom [34].

The Ca^2+^ antagonists verapamil, diltiazem, and nifedipine have all been shown to reduce the cardiotoxicity of venom from *Physalia physalis* (hydrozoan) [35], *Carybdea rastonii* (cubozoan) [36,37], *Chironex fleckeri* (cubozoan) [38], and *Cyanea capillata* [39]. Another study, however, found that verapamil did not affect Ca^2+^ influx, but it was inhibited by La^3+^, a non-specific channel and pore blocker. As such, if the Ca^2+^ channels were blocked and the Ca^2+^ still entered the cell, it can be inferred that the venom creates a bypass to this system, such as a non-selective pore, which cannot be blocked by verapamil. This supports the presence of a pore-forming toxin in the venom of *Chironex fleckeri* [40], and lends credence to the notion that the mechanism of extracellular Ca^2+^ entrance is dominated by non-specific translocation through pores in the cell membrane. More than ten hemolytic proteins have been isolated from jellyfish venom thus far. These new proteins have been shown to operate as non-selective cation pore-forming proteins, hence contributing to extracellular Ca^2+^ entry [24,41,42,43,44].

*Chironex fleckeri* venom was also shown to cause extracellular Na^+^ entry and intracellular Ca^2+^ overload in cardiomyocytes. This effect was not hindered by Ca^2+^ or Na^+^ channel blockers or inhibitors of Na^+^/H^+^ or Na^+^/K^+^ ATPase exchange but was blocked by prior exposure to a solution containing no Na^+^ and by Ni^2+^. This supports a possible role of the Na^+^/Ca^2+^ exchange on the Ca^2+^ overload [34]. Another study found that *Chiropsalmus quadrigatus* (cubozoan) venom exhibits both vasoconstrictor and cardiodepressive effects in rabbits and associated that effect with the activation of voltage-dependent Ca^2+^ channels and consequent Ca^2+^ overload [45].

### 5.2. Induction of Na^+^ and K^+^ Currents

The venom of *P. noctiluca* (scyphozoan) nematocysts can induce an ionic current, mainly Na^+^, through the plasma membrane, most likely due to a pore-forming process. The hemolytic and cytolytic action of *P. noctiluca* venom is presumably due to this NaCl inflow followed by water and subsequent cell enlargement. Amiloride, an Na^+^ channel blocker, can inhibit this effect. It has been demonstrated that this venom is thermolabile, having its hemolytic activity significantly reduced above 40 °C and abolished after boiling. It is known that heat exposure changes the three-dimensional structure of proteins, inducing their loss of activity. As such, this result supports the hypothesis of a protein component of the venom being involved in pore formation [46]. A zinc metalloproteinase has been identified on *P. noctiluca* venom, which contains a ShK toxin domain. This neurotoxin inhibits K^+^ channels and is probably involved in sting toxicity [47]. Similar metalloproteinases have also been described in *Rhopilema esculenta* (scyphozoan)*, Cyanea nozaki* (scyphozoan), *Nemopilema nomurai* (scyphozoan)*,* and *Aurelia aurita* (scyphozoan) [48]. The metalloproteinases identified exhibited caseinolytic, gelatinolytic and fibrinolytic activity, and seem to give a significant contribution to the toxic effects of jellyfish venom. 

### 5.3. Targeting the Adrenergic System

The adrenergic system also seems to play a role in the cardiovascular toxicity of jellyfish venom. This was first demonstrated when the aorta contraction induced by *Carybdea rastonii* (cubozoan) venom was inhibited by trifluoperazine and phentolamine [37]. Further research revealed that prazosin and propranolol reduced the tachycardia and venom-induced pressor response in anesthetized rats [49,50]. These results, however, were not consistent between jellyfish species. 

Propranolol, but not prazosin, significantly reduced the concentration-dependent inotropic response in the left atria elicited by *Malo maxima* (cubozoan) venom [51]. However, previous treatment of prazosin did not significantly reduce hypertension or prevent cardiovascular collapse caused by the sting of the jellyfish *Chiropsalmus* sp. (cubozoan) [52]. Prazosin had no discernible impact on the cardiovascular effects, but greatly reduced the pressor response to tentacle extract from *Chironex fleckeri* [53]. Another investigation on *Chironex fleckeri* venom found that propranolol and prazosin have no effect on the cardiovascular toxicity of this venom [54].

In another investigation, *Cyanea capillata* venom caused intracellular Ca^2+^ overload via intracellular Ca^2+^ release on mouse cardiomyocytes. Esmolol, atenolol and propranolol were able to inhibit this effect. Both cyclic adenosine monophosphate concentration (cAMP) and protein kinase A (PKA) activity increased proportionally, showing that beta-adrenergic signaling is involved in the cardiotoxicity of jellyfish venom. The significant extracellular Ca^2+^ influx through pore-forming components, bypassing the adrenergic mechanism of Ca^2+^ overload, may explain previous unfavorable results with adrenergic blockers. This hypothesis is supported by the fact that, while in the absence of extracellular Ca^2+^, propranolol completely inhibits the Ca^2+^ overload, this inhibition is only partial when the cardiomyocytes were placed in a solution containing Ca^2+^ [55]. *O. sambaquiensis* (hydrozoan) and *Chiropsalmus quadrumanus* extracts were found to strongly interfere with noradrenergic neurotransmission of the rat vas deferens without affecting purinergic response or smooth muscle structure [56].

### 5.4. Endothelial Nitric Oxide Synthase (eNOS) Induction

Endothelial nitric oxide synthase (eNOS) induction by *Cyanea capillata* tentacle extract leads to nitric oxide (NO) production in a dose and time-dependent manner. Studies revealed that the extract caused the phosphorylation and activation of eNOS mostly via phosphatidylinositol 3-kinase/protein kinase B (PI3K/Akt)-dependent, protein kinase C/inositol trisphosphate receptor (PKC/IP3R)-sensitive, and Ca^2+^-dependent pathways. These results confirm that the jellyfish venom-induced vasodilation is mediated by NO release via the stimulation of eNOS in endothelial cells [57].

### 5.5. Tubule Length of the Nematocyst and Severity of the Pain

Recent data suggest that the variation in pain severity could be attributed to the tubule length of the nematocyst, this way causing variable epithelial lesions. Kitatani et al. [58] found that nematocyst tubules from the dangerous jellyfish species *Carybdea brevipedalia* (cubozoan), *Chrysaora pacifica* (scyphozoan), and *Chironex yamaguchii* (cubozoan) are long enough (>200 m) to pierce the human epidermis and stimulate pain receptor fibers’ free nerve ends. Less harmful species like *Aurelia aurita*, on the other hand, have shorter tubules [58]. While the primary noxious stimulus may be triggered by the tubule penetration, which stimulates pain receptor neurons, the persistent pain may be due to venom injected into the skin. In fact, the jellyfish venom itself activates the transient receptor potential vanilloid-1 (TRPV1) non-selective cation channels present in nociceptive neurons, therefore causing pain [59,60].

### 5.6. Hemostasis Disturbance

*Rhizostoma pulmo* (scyphozoan) venom was found to be able to influence the hemostatic system on three separate levels, exhibiting fibrinolysis, fibrinogenolysis, and suppression of ADP-induced platelet aggregation. It also demonstrated considerable hemolytic activity against human red blood cells, as well as high proteolytic activity against substrates such as (azo) casein and gelatin. However, EDTA (metalloproteinase inhibitor) reduced this proteolytic activity but not PMSF (serine proteinase inhibitor). Rhizoprotease, a new metalloproteinase of 95 kDa, was isolated in the venom [61]. *Nemopilema nomurai* (scyphozoan) venom contains a chymotrypsin-like serine protease with fibrinolytic activity [62]. *Aurelia aurita* shows strong fibrinogenolytic activity [63].

### 5.7. Other Reported Mechanisms of Toxicity

The venom of *Nemopilema nomurai* (scyphozoan) can induce significant edema. This venom does not seem to operate as an acute proinflammatory agent, but rather it plays a role in the persistence of inflammation. Its effect on edema is mostly ascribed to its influence on vascular permeability via a mechanism of direct degrading basement membrane components. These findings support the use of antihistaminic drugs in scyphozoan stings. Phospholipase is not involved in the vasopermeability shift generated by *Nemopilema nomurai* venom [64]. *Pelagia noctiluca* and *Cyanea capillata* have an oxidative effect, inducing inflammation and apoptosis [28,65,66,67]. Phospholipase A_2_ plays an important role in inflammatory pathways and is an active component of some animal venoms. Nevalainen et al. [68] found phospholipase A2 in cnidarian venom and suggested it may play a role in its toxicity. Histamine release is induced by *Physalia physalis* venom by fast, short-duration exocytosis of granules and lengthier lysis of mast cells [69]. *Cyanea capillata* venom was also found to induce histamine release [70].

### 5.8. Delayed Reactions

A type IV hypersensitivity reaction triggered either by a sequestered antigen persisting in the skin or by a cross-reacting antigen in the venom is postulated to be in the genesis of the delayed, persistent reactions after jellyfish stings [71].

### 5.9. Irukandji Syndrome

*Carukia barnesi* venom can activate neural Na^+^ channels, inducing catecholamine release and vasoconstriction. This mechanism seems to be accountable for the sympathomimetic-like symptoms of Irukandji syndrome, namely hypertension, and tachycardia [50]. Like *Carukia barnesi, Malo maxima* venom activates a neural sodium channel, releasing endogenous noradrenaline and calcitonin gene-related peptide antagonist (CGRP) [51]. The following mechanisms have been implicated in acute heart failure: (i) stress-related cardiomyopathy caused by hypertension or direct cardiomyocyte toxicity of catecholamines [72], and (ii) pore formation on cardiomyocytes, which disturbs cellular function and permits poisons to enter and cardiac enzymes to exit [73].

## 6. Envenomation Syndromes

As mentioned above, the toxic effect of jellyfish venom varies greatly. The different classes of jellyfish tend to cause different syndromes, although most signs and symptoms are nonspecific and shared between classes.

### 6.1. Class Scyphozoa

Scyphozoans (true jellyfish) are abundant and ubiquitous. Although responsible for most jellyfish envenomations worldwide, only a very small fraction of these are severe, rendering them less dangerous than the other jellyfish classes [14]. Symptoms related to this envenomation are compiled in Table 1. Upon contact of the skin with the jellyfish, pain of variable intensity is the most frequent and often the only clinical manifestation of the envenomation [13]. A vesiculourticarial eruption may appear, possibly resembling the shape of the tentacles or the bell of the offending animal. Edema, pruritus, hemorrhage, or necrosis may be present as well. Even though erythema and pain typically subside in hours to days, sequelae of the sting may persist, namely scaring and hyperpigmentation [27]. In some cases, the eruption may become generalized, persistent, or be delayed [74]. Systemic symptoms, even though rare, may occur. Lethal cases have been reported [75]. 

### 6.2. Class Hydrozoa

*Physalia physalis*, the most prominent member of this class, is a colonial organism that has a gas bladder and numerous tentacles made up of cell populations with diverse roles. Morphologically, it has a vivid color that ranges from green to violet [150]. Hemolytic, cardiotoxic, and neurotoxic activities of *Physalia* venom have been demonstrated. Typical lesions develop immediately after contact with the organism, including linear urticariform plaques, as well as severe pain. Systemic symptoms may arise, such as nausea and vomiting, cold sweats, syncope, heart arrhythmia, and even death. Furthermore, allergic reactions to the venom might cause anaphylactic shock, which can result in death within minutes [150,151]. Rhabdomyolysis and acute renal failure have also been described [122].

### 6.3. Class Cubozoa

The members of this class, also known as box jellyfish, are one of the most hazardous marine organisms in the world [50]. Even though they cause a smaller number of envenomations, compared to the other classes, most encounters result in severe symptoms. This class encompasses two families, Chirodropidae (such as *Chironex fleckeri*) and Carybdeidae (to which belong the species most associated with Irukandji syndrome). Their venom is cardiotoxic, hemolytic, dermonecrotic, and neurotoxic [152]. Envenomations from these organisms can result in cardiorespiratory depression and death within minutes [34,73,153,154]. Skin necrosis has also been described [82]. A delayed soft tissue necrosis might occur, which is thought to be caused by an immune response to the tubules lingering in the lesion or by the triggering of undischarged nematocysts [19]. 

#### Irukandji Syndrome

Irukandji syndrome is a severe illness produced by the envenomation of some species of small jellyfish from the Cubozoa class [155]. It consists of a clinical picture dominated by systemic symptoms similar to a catecholamine surge, including hypertension, tachycardia, intense pain, and muscle cramping, eventually leading to pulmonary edema, shock and cerebral hemorrhage [156]. So far, the species implicated in the syndrome are *Carukia barnesi* [102], *Alatina mordens, Carybdea alata, Malo maxima, Carybdea xaymacana* [157], *Morbakka fenneri*, *Malo kingi, Carukia shinju, Gerongia rifkinae* [33], *Alatina reinensis, Gonionemus oshoro* [16] and *Alatina alata* [158]. However, species identification is not necessary to diagnose Irukandji syndrome.

The first cases described happened in the northern Australian territories [159]. However, similar disorders have been observed all over the tropical waters, including Thailand, the Caribbean, Florida, and Hawaii, although not all instances have been linked to a specific species [103,104,105,156,160,161]. Stings seem to occur in short, pandemic outbreaks, so patients often present themselves to the hospital in clusters [162]. 

The offending jellyfish often goes unnoticed, but sufferers frequently experience severe pain at the afflicted region after the triggering incident. Erythema, edema, and tentacle marks may be present [163]. The initial local discomfort is generally mild and subsides in less than half an hour. During this time, an erythemato-papular skin lesion of around 2 cm, the dimension of the offending animal, might appear, popularly referred to as “goose pimples”, which normally dissipates quickly but can last for days [16]. The onset of severe systemic symptoms, the cornerstone of the typical Irukandji syndrome, can range from 5 to 120 min, but most often takes 30 min [164]. Muscle cramps, serious back, thoracic, and abdominal pain, nausea, vomiting, diaphoresis, anxiety, restlessness, headache, localized sweating, and piloerection are the main symptoms [73,159,165,166]. The condition is often linked to a sense of “impending doom” [157]. Because the toxin is hyperadrenergic, hypertension and tachycardia are common. There have been reports of blood pressures as high as 300/180 mmHg [167]. Pallor, peripheral cyanosis, oliguria, tremor, and cerebral edema have also been described [168]. Ventricular tachycardia, myocardial injury [72,106], cardiomyopathy with abrupt pulmonary edema [147,169] and cardiogenic shock [32,156] may occur. In extreme cases, ventilatory failure may ensue, necessitating admission to an intensive care unit [32]. Pancreatitis, priapism, and acute renal failure were also associated with this syndrome, notwithstanding their rarity [156]. 

Although exceedingly painful and frequently necessitating narcotic analgesia and inpatient hospitalization, Irukandji syndrome is typically not deadly, especially if supportive care is given early [156]. Most patients who seek emergent care following a sting can be returned home in the same day. Besides, not all encounters with species capable of producing Irukandji syndrome result in this clinical state [170,171]. Even though the pain only lasts for some hours, there are reports of pain recurrence, necessitating additional hospital visits [156]. In one reported case, the pain recurred up to a year later [104]. The first described fatal Irukandji disease cases were caused by intracranial hemorrhage [148,149]. These are thought to have occurred because of extreme hypertension.

### 6.4. Seabather’s Eruption

Seabather’s eruption is a severely pruriginous papule-erythematous dermatitis that arises following exposure to marine water in areas of the body covered by bathing clothes. It is caused by the larval form of *Linuche unguiculata*, which becomes trapped in the bathing suit and releases its toxin [172,173]. Lesions usually occur in the gluteal region, the most afflicted areas among surfers. However, the chest, belly, arms, and thighs, which were in direct contact with the surfboard, activated the larvae nematocysts [174]. Lesions could also be found in flexural regions. Chills, fever, nausea, vomiting, diarrhea, headache, and abdominal pain are found on rare occasions and usually occur in children or cases of severe envenomation [76,175]. 

### 6.5. Delayed Reactions

Jellyfish stings commonly result in immediate local skin reactions, typically characterized by erythema, edema, vesicles, and severe localized pain. However, in rare cases, the patients may present with delayed allergic reactions with similar symptoms days or months after the sting, even without having experienced an immediate reaction. While a toxic mechanism is held accountable for the immediate reaction, the delayed eruptions are immune-mediated [83,90]. Delayed cutaneous reactions can take multiple presentations, such as keloid-like plaques [71], nodular and papular eruptions [84,85,139,176], linear pigmentation [86], vesicles [177], and granulomatous infiltration [87].

### 6.6. Eye Lesions

Jellyfish venom has toxic effects on ocular tissue. A sting to the eye may result in pain, photophobia, conjunctival injection, punctate epithelial keratitis, iritis, persistent mydriasis, peripheral anterior synechiae, corneal stromal edema, foreign body sensation, and increased intraocular pressure, and usually resolves without sequelae [100,178,179,180,181]. However, severe fundus lesions have also been described, namely retinal vascular occlusion, thinning of the retina, optic atrophy, and scar formation in the macular area [182]. Table 1 compiles the various symptoms related to jellyfish envenomation reported in the literature.

## 7. Diagnosis

The diagnosis of a jellyfish sting is predominantly clinical. Some clinical characteristics of the lesion may raise suspicion as to which cnidaria class caused the envenomation. If a cnidarian sting is suspected but the patient cannot recall having contact with a jellyfish, a skin scrape/sticky tape test may reveal nematocysts in the lesion. Dermoscopy, histology, and reflectance confocal microscopy can be useful as well.

### 7.1. Signs and Symptoms

**Figure 1:**
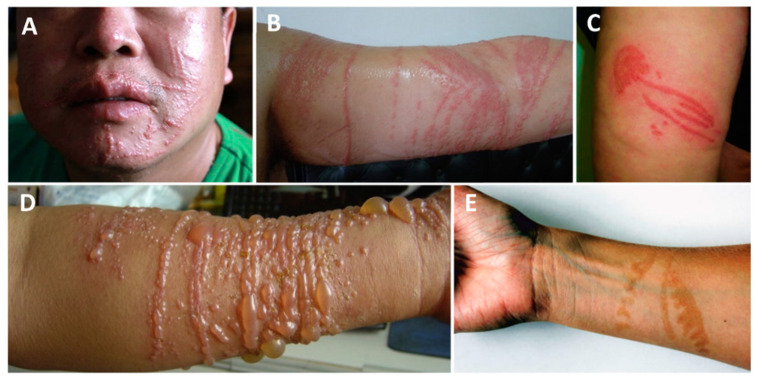
Scyphozoan jellyfish stings: (**A**,**B**)—multiple linear erythematous beaded marks of a *Cyanea nozakii* sting; (**C**)—confluent erythematous papules in the shape of a jellyfish; (**D**)—linear, vesicular lesions after a *Stomolophus meleagris* sting; (**E**)—hyperpigmentation two months after the contact with a *Pelagia noctiluca* jellyfish. (**A**,**B**) reproduced from [183], (**C**) from [184], (**D**) from [132], (**E**) from [78].

Scyphozoan stings can take a multitude of appearances (Figure 1). Unlike other classes, these species may leave the “imprint” of their bodies on the skin, giving rise to a jellyfish-like erythematous lesion. The tentacle marks can be flat, edematous, papular, and vesicular. Since these organisms are ubiquitous, a scyphozoan sting should be suspected when a patient has a history of sea bath and comes in with a variably painful, erythematous, linear, or jellyfish-shaped lesion.

**Figure 2:**
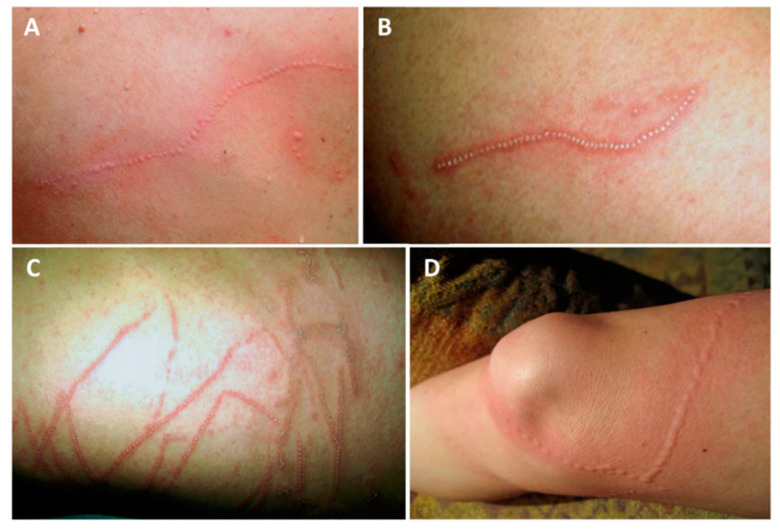
*Physalia physalis* jellyfish sting marks: (**A**)—typical linear, beaded tentacle marks, 2 h after a sting on the dorsum; (**B**)—12 h after a sting on the dorsum, showing the “frosted” appearance; (**C**)—12 h after a sting to the chest; (**D**)—beaded, urticariform eruption following a sting. (**A**–**C**) reproduced from [133], with permission.

Regarding the class Hydrozoa, upon contact with *Physalia physalis* tentacles, an immediate, painful skin rash will appear (Figure 2). The severity of the lesion ranges from erythematous urticarial linear beaded plaques to vesiculobullous eruptions. On some occasions, the lesion may blister or even become necrotic. Lesions may take a “frosted” appearance due to superficial skin necrosis. A sting from this species should be suspected when a sea bather reports intense pain and a linear rash with a “string of beads” appearance.

**Figure 3:**
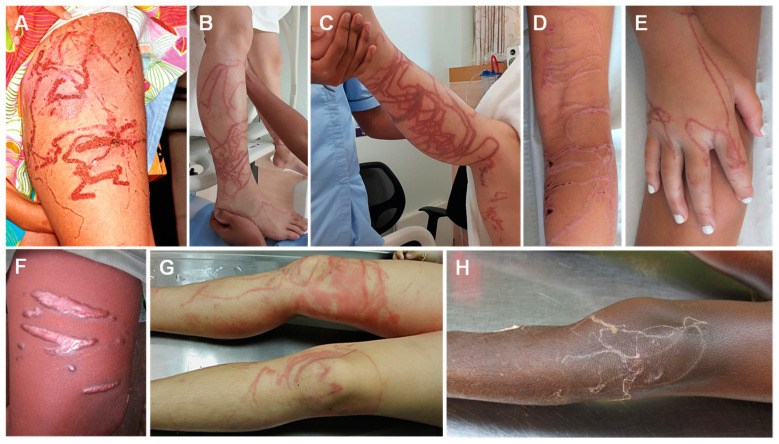
Cubozoan jellyfish stings: (**A**)—*Chironex fleckeri* sting; (**B**,**C**)—near-fatal cubozoan sting on a 31-year-old man; (**D**,**E**)—tentacle-shaped skin necrosis on two children stung by *Chironex sp*.; (**F**)—scar on the thigh of a 28-year-old Thai man, 6 months after a sting; (**G**)—erythematous urticarial lesions following a fatal *Chironex fleckeri* sting; (**H**)—pale, serpiginous tentacles adherent to a 6-year-old boy after a fatal *Chironex fleckeri* sting. (**A**) reproduced from [186], (**B**,**C**) from [187], (**D**,**E**) from [142], (**F**) from [141], (**G**) from [188] and (**H**) from [143], with permission.

Cubozoan stings leave relatively wide, ladder-like, cross-hatched marks, resembling those of a whip (Figure 3). They often have a “frosted” appearance due to superficial skin necrosis [185]. 

**Figure 4:**
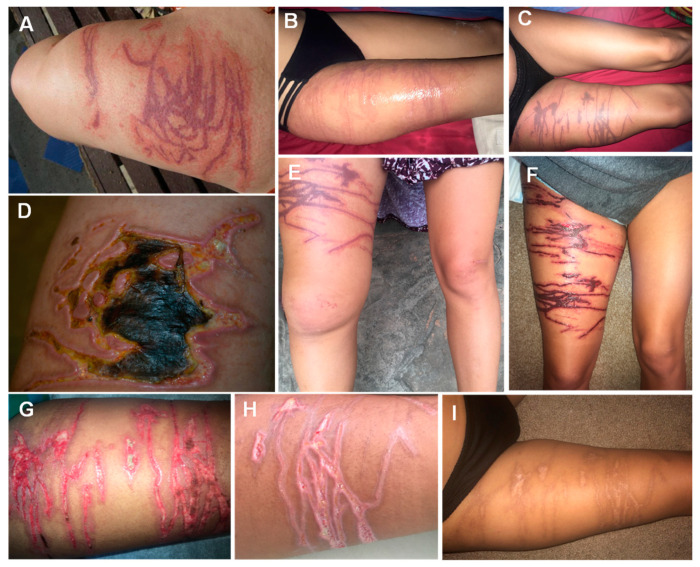
Evolution of Cubozoan jellyfish stings: (**A**)—brownish, erythematous tentacle marks a few hours after a cubozoan sting; (**B**)—some weeks after the envenomation, the wound showed signs of infection and gangrene; (**C**)—erythematous, linear marks on the day of the envenomation; (**D**)—day 2, dark purple marks corresponding to the tentacle contact sites—right leg edema; (**E**)—day 4, worsening edema of the right leg; (**F**)—day 12, scab formation with exposure of textured granulation tissue underneath and serous exudate; (**G**)—day 19, increased granulation tissue, the wound edges have started to re-epithelize; (**H**)—day 24, the wound is healing by secondary intention; I)—wound appearance 120 days after the sting. (**A**,**B**) reproduced from [77], (**C**–**I**) from [141], with permission.

The lesions may complicate with necrosis and take several weeks to fully heal (Figure 4). A cubozoan sting should be suspected in a patient bathing in Indo-Pacific shallow waters that presents with severe pain, skin marks as described above, and possibly with distressing systemic symptoms.

**Figure 5:**
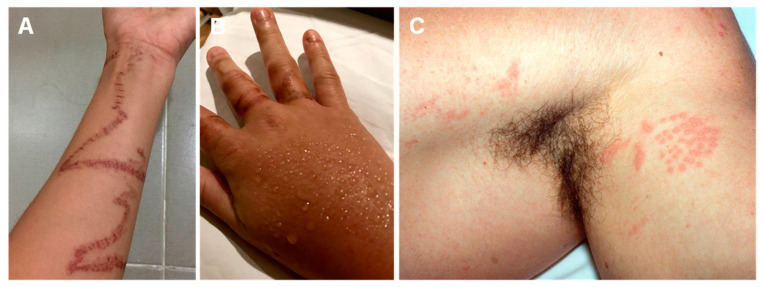
Irukandji jellyfish stings: (**A**,**B**)—*Morbakka *spp., a possible agent of Irukandji syndrome, can leave a caterpillar track mark on the site of the sting; (**B**)—localized sweating can be found on sting sites; (**C**)—”Goose pimples” appearance on the contact site. (**A**,**B**) reproduced from [108], (**C**) from [162], with permission.

Irukandji syndrome occurs mostly in the Indo-Pacific region. *Morbakka *spp., a possible agent of Irukandji syndrome, can leave a caterpillar track mark on the site of the sting [108]. However, most Irukandji stings leave only “goose pimples” or no mark at all. Localized sweating can often be seen (Figure 5). When Irukandji syndrome is suspected, the clinician should bear in mind the following differential diagnosis: other cnidaria stings, hyperthyroidism, sympathomimetic toxicity, pancreatitis, pheochromocytoma, rhabdomyolysis, anaphylaxis, acute decompensated heart failure, acute coronary syndrome, and decompression illness [155].

**Figure 6:**
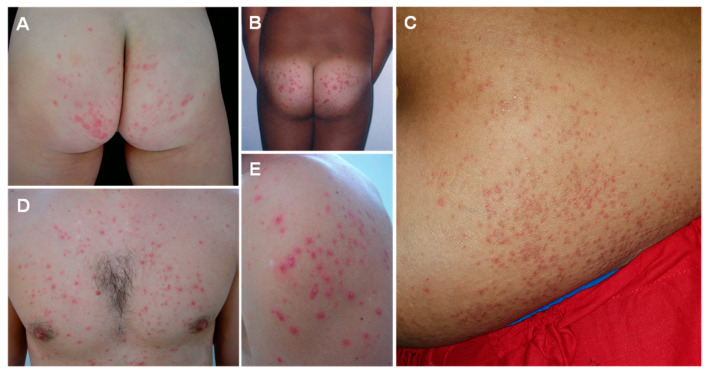
Seabather’s eruption caused by jellyfish stings: highly pruritic papules and pustules on the gluteal region (**A**,**B**), abdomen (**C**), thorax (**D**) and shoulder (**E**), corresponding to friction areas covered by the bathing suit. (**A**) reproduced from [175], (**B**) from [190], (**C**) from [191], (**D**) from [175], (**E**) from [191], with permission.

Seabather’s eruption should be suspected in a patient bathing in the Atlantic coast of Central and South America, and the Atlantic African coast, from Mauritania to Gabon, presenting with a highly pruritic papular rash in the areas covered by the bathing suit (Figure 6). Seabather’s eruption can be confused with swimmer’s itch, which occurs after bathing in freshwater and is found all over the world. Swimmer’s itch affects only exposed regions of the body, and the agents responsible are *Schistosoma *spp. cercariae [174]. Insect bites and scabies are other prominent differential diagnoses for swimmer’s itch [189].

**Figure 7:**
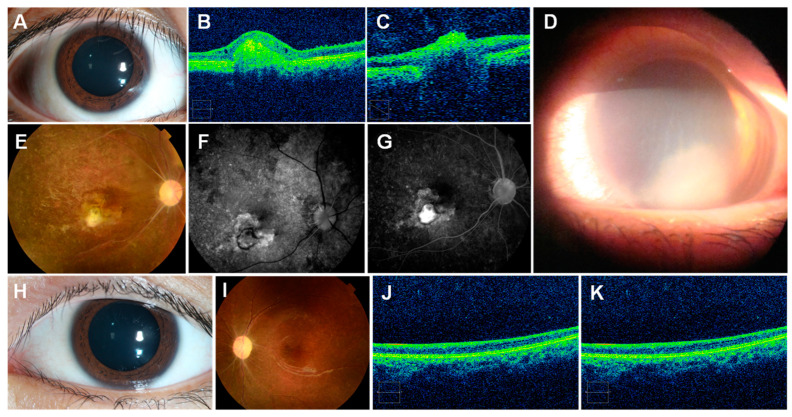
Jellyfish stings to the human eye: (**A**)—injected conjunctiva following an ocular sting; (**B**)—optical coherence tomography shows elevated lesion and thinning of the retina; (**C**)—severe thinning of the retinal nerve fiber layer; (**D**)—three days after an ocular sting, showing marked corneal edema with an area of diffuse severe keratitis occupying the lower half of the cornea; (**E**)—fundoscopy, revealing optic disc pallor, retinal vascular occlusion, retinal pigmentation, scar; (**F**,**G**)—fundus fluorescein angiography; (**H**)—normal examination; (**I**)—pale optic disc, retinal vascular occlusion, pigmentation of the retina; (**J**,**K**)—thinning of the retinal layer. (**A**–**C**,**E**–**K**) reproduced from [182], (**D**) from [192], with permission.

After a sting to the eye, conjunctival edema, epithelial corneal defects, and foreign bodies may be seen [178] (Figure 7).

### 7.2. Medical Exams

**Figure 8:**
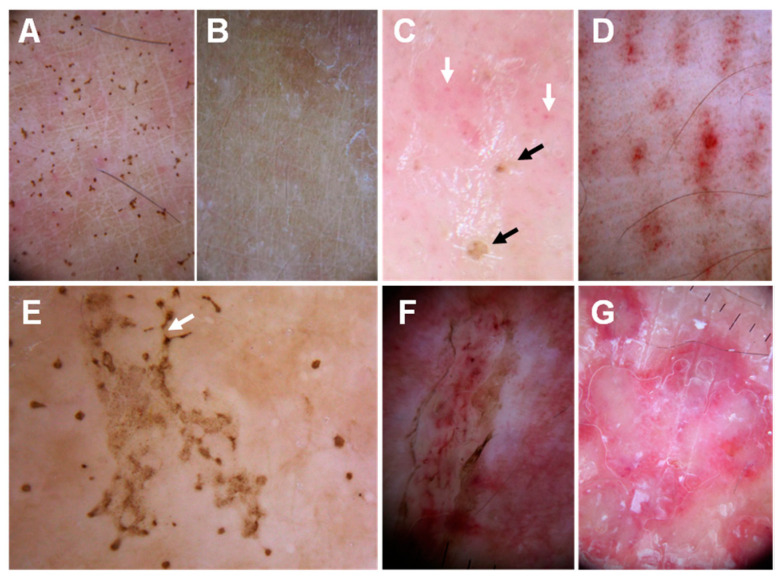
Dermoscopic findings in *Pelagia noctiluca* jellyfish stings: (**A**)—brown dots on a 1-week-old sting; (**B**)—21 days after the sting, the brown dots have completely disappeared; (**C**)—pinpoint brown crusts (black arrows) and red dots (white arrows) in a pink background; (**D**)—‘Linear purpura’: linear bands composed of red dots regularly spaced in a tabby pattern; (**E**)—‘Chinese characters’ pattern: brown dots connected by light brown granular lines (white arrow), matching a linear blistered lesion; (**F**)—‘Serpentine ulceration’. Scales and brown dots delimit the edges of the ulcer, with linear purpura inside; (**G**)—‘Circular milky-red areas’ corresponding to a recurrent, persistent, inflammatory reaction to a sting. Reproduced from [136], with permission.

Dermoscopic findings may be species-specific and represent a diagnostic tool of jellyfish sting. A study on the dermoscopy of *Pelagia noctiluca* stings identified four dermoscopic features: brown dots, brown ‘Chinese characters’ pattern, pinpoint brown and whitish-yellow crusts. When a clear history of interaction with the cnidarian is unavailable, observation of these dermoscopic characteristics in typical cases of *Pelagia noctiluca* stings may aid the diagnosis [136] (Figure 8).

**Figure 9:**
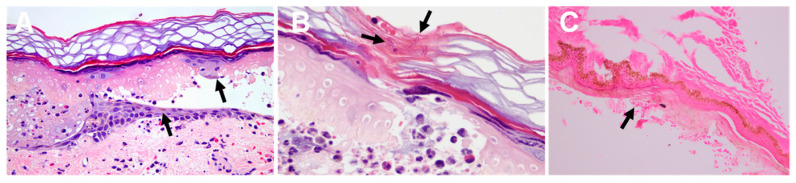
Histology of jellyfish stings: (**A**)—epidermal necrosis and vesicle formation following a *Pelagia noctiluca* sting. Pigmented keratinocytes (black arrows), vasodilatation, edema, and erythrocyte extravasation are displayed; (**B**)—fragments of nematocyst tubules are shown in the stratum corneum (black arrow); (**C**)—remains of nematocysts (arrow) following a *Chironex fleckeri* sting. (**A**,**B**) reproduced from [136], and (**C**) from [143], with permission.

Histology of the lesions may demonstrate the presence of nematocysts, as well as inflammation signs (Figure 9).

**Figure 10:**
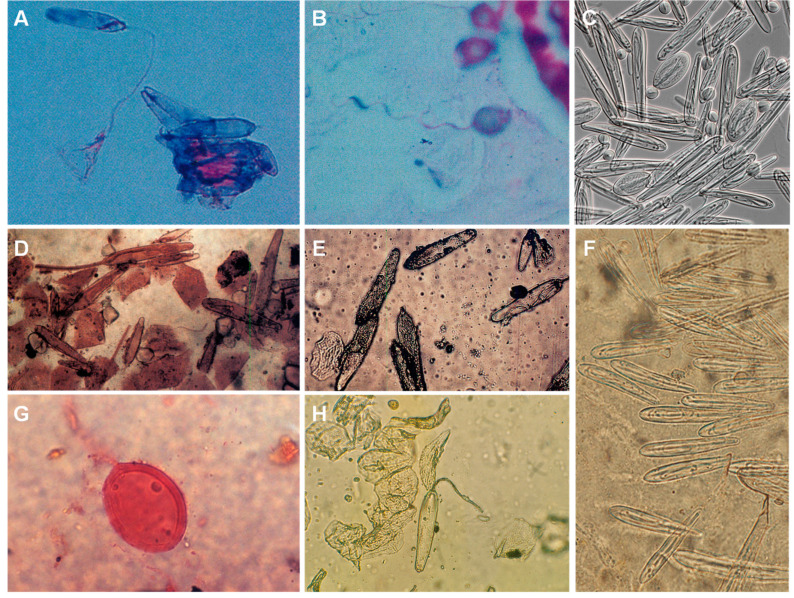
Jellyfish nematocysts retrieved from human skin: (**A**)—ruptured nematocyst in a skin scraping from a *Chiropsalmus quadrumanus* sting lesion; (**B**)—nematocyst from *Physalia physalis* jellyfish; (**C**)—nematocysts isolated from *Chironex fleckeri* tentacles; (**D**)—*Chironex fleckeri* nematocysts (skin scraping); (**E**)—*Chironex fleckeri* nematocysts (sticky tape); (**F**)—nematocysts harvested from *Chironex* spp. tentacles with sticky tape; (**G**)—skin scraping from a patient with Irukandji syndrome (species is unknown); (**H**)—sticky tape from a *Chironex fleckeri* sting. (**A**,**B**) reproduced from [194], (**C**) from [44], (**D**,**E**) from [195], (**F**) from [196], (**G**) from [156] and (**H**) from [143], with permission.

Nematocyst identification on skin scrapings/sticky tape confirms the occurrence of a jellyfish sting [193]. Furthermore, as nematocyst morphology is species-specific, a skin scraping may help identify the species responsible for the envenomation or, at least, the class of the jellyfish [170] (Figure 10). 

The radioallergosorbent test (RAST), enzyme-linked immunoabsorbent assay (ELISA), and the Ouchterlony immunodiffusion test are useful for detecting allergic responses to unnoticed cnidarian encounters. By detecting antibodies against jellyfish, these may also identify people who have become sensitized during a previous encounter with these animals and, therefore, are at risk of developing a more severe reaction following a future interaction [15,197].

Color Doppler Ultrasonography (CDUS) has been used for evaluating the therapeutic response in delayed allergic reactions to cnidarian stings. In all described cases, the initial evaluation portrayed dermal thickening and decreased echogenicity, when compared to that of healthy individuals. It is worth noting that CDUS enabled clinicians to determine that the cutaneous inflammation had subsided and, as a result, discontinue the therapy, even when the clinical examination of the lesions remained nearly unaltered. The Doppler mode revealed hypovascularization, which could be attributed to vasoconstriction caused by jellyfish stings. Color or power Doppler imaging is essential for ruling out vascular contraction diseases that could lead to ischemia, namely severe vasospasm caused by jellyfish envenomation [83].

Seabather´s eruption: although the histological aspect of the biopsy is not specific, it may aid the differential diagnosis by displaying superficial and deep interstitial and perivascular infiltrates composed of eosinophils, neutrophils, and lymphocytes. The ELISA (enzyme-linked immunosorbent assay) technique allows for the detection of IgG antibodies to *Linuche unguiculata* in afflicted people’s serum [175].

## 8. Treatment

The most significant intervention is primary prevention. Many places have created information, warnings, and beach closures as a result of local observations and climate analyses [198]. A full-body lycra “stinger suit” can prevent some envenomations, particularly those caused by the smaller Irukandji jellyfish [162]. Secondary prevention, such as the use of sunscreen lotion containing jellyfish sting inhibitors, has been shown to lessen symptoms following jellyfish exposure [199,200]. The initial approach to a jellyfish envenomation should be guided by the following steps:

(a) Patient stabilization—victims should be removed from the water to prevent further stinging and drowning. If needed, life support maneuvers should be performed. The victim should be brought to emergency care if needed;
(b) Preventing nematocyst discharge—care should be taken to minimize the venom load by preventing further discharge of the nematocysts. As such, vinegar is usually employed to prevent the discharge of the remaining nematocysts, but this use is based largely on empirical knowledge. Research regarding this use is conflicting. It is generally advised to use vinegar in Hydrozoan and Cubozoan stings and to avoid its use on Scyphozoan. Ballesteros et al. showed that vinegar leads to *Pelagia noctiluca* nematocysts discharge in vitro [201]. However, the most recent review on this topic suggested vinegar to be beneficial in *Cyanea capillata* and *Pelagia noctiluca* envenomation, so further research is needed [14,202]. After inactivating the nematocysts, adherent tentacles must be removed gently, using tweezers or a similar tool. Tentacle removal can also be attempted by rinsing with seawater [203], however, some studies recommend against this practice, as it can induce further venom delivery [202]. Freshwater must not be used for rinsing, as the osmotic challenge would induce nematocyst discharge [119]. An in vitro study found that lidocaine might block nematocyst discharge from *Pelagia noctiluca* [25]. Moreover, topical lidocaine was demonstrated to both reduce pain and prevent nematocyst release [25].
(c) Alleviating local venom effects—heat has shown significant pain relief for many jellyfish stings, particularly those caused by *Physalia physalis* [160,204,205].
(d) Controlling systemic effects—analgesics, antihistamines, corticosteroids, anti-hypertensive drugs are some of the treatments that may be required, depending on the symptoms;
(e) Preventing complications—if a sting is at risk of becoming infected, antibiotics should cover *Streptococcus* spp., *Staphylococcus* spp., and marine organisms, such as *Vibrio* spp. According to Center for Disease Control recommendations, tetanus prophylaxis is indicated whenever there is a minor wound, and the patient is not up to date with the tetanus vaccination scheme. As such, tetanus prophylaxis must also be considered [6,76,206].

### 8.1. Particularities of Each Class

Regarding scyphozoan stings, most cases are mild, and management usually includes oral or topical antihistamines and topical corticosteroids. Systemic corticosteroids may be required in severe cases. Analgesics (acetaminophen, non-steroidal anti-inflammatory, opiates) and topical antibiotics can be useful to control pain and prevent or treat infections [13].

In hydrozoan stings, only a small fraction of nematocysts discharge on initial contact. As such, tentacles should be removed carefully, ideally after being doused with an inactivating product. A recent study [207] showed that, while different kinds of vinegar prevented discharge in *Physalia* spp., even a minor dilution of vinegar significantly diminished inhibitory effects or resulted in partial nematocyst discharge. In addition, a 30 s vinegar irrigation is enough to decrease stinging. The suppression of discharge was not solely a function of pH, as this effect was not obtained with different acidic solutions. Alcohols promoted discharge, resulting in enhanced hemolysis, and should therefore be avoided. Finally, the findings confirm prior research [204,208,209] that supports the use of heat in the treatment of *Physalia* spp. stings, since heat greatly decreased hemolysis. The use of cold not only failed to diminish hemolysis but also exacerbated stinging. The only systematic review concerning pain relief following *Physalia* spp. stings revealed that hot water, compared to ice packs, was capable of a ≥50% pain reduction after 20 min of treatment, with a number need to benefit of 1.8 (1.4 to 2.7, CI 95%) [208]. 

In the case of cubozoan stings, the venom load released into the sting site is linked to tentacle contact length and sequelae severity. Because only about 1% of nematocysts discharge upon initial contact [210], successful and careful removal of clinging tentacles is critical in the treatment of potentially fatal cubozoan stings, as ineffective removal of adherent tentacles has the potential to significantly aggravate sting results by increasing the venom load [211]. Vinegar has been proven in vitro to be a powerful, irreversible inhibitor of cubozoan nematocyst discharge [210,211,212]. Some case series show improved outcomes, including a better chance of survival, when vinegar is used in first aid [80,187]. A recent in vitro study illustrates that rinsing the sting with sea water, scraping tentacles away, and using cold packs can exacerbate venom-induced hemolysis, a measure of sting sequelae, in the cubozoans studied. Furthermore, its findings support the use of vinegar or the commercial over-the-counter spray Sting No More^TM^ (composed of vinegar, urea, magnesium sulfate, and copper gluconate) before tentacle removal as they were effective in deactivating the nematocysts. Besides, the authors suggest that the sting site should be immersed in 45 °C water or a 45 °C hot pack for 45 min after the removal of the tentacles, as it inactivates the hemolytic activity of the venom [211]. Other small studies support heat application [213]. However, in a small randomized controlled trial [214], hot water immersion only offered mild relief from the acute pain of *Chironex fleckeri* stings, and its performance was similar to ice pack application. This is surprising given the proven benefit of hot water on *Physalia* spp. stings. The lack of effect of the heat might be attributed to the fact that, in this study, treatment was postponed until the patient arrived at the emergency department. As a result, the effect of heat was probably symptomatic rather than venom inactivation. A *Chironex fleckeri* antivenom is available and should be administered as soon as possible to a suspected *Chironex fleckeri* victim in the following circumstances: cardiorespiratory instability, unconsciousness, airway or ventilation compromise, severe pain, and the possibility of significant skin scarring [215].

### 8.2. Irukandji Syndrome

As with most jellyfish stings, first aid includes removing the victim from the water and liberally dousing the sting site with vinegar. It has been demonstrated that vinegar irrigation can improve the outcomes of Irukandji victims [19,108,119,203,210]. The commercial copper gluconate-based product Sting No More™ has also revealed its merit in the prevention and first aid treatment of Irukandji syndrome [210].

This syndrome is, most of the time, extremely painful, often requiring opioids [155,216]. All patients with Irukandji syndrome must have active pain management, which includes regular pain assessments using a validated tool [106].

Because of the possible cardio-respiratory consequences of envenomation, patients’ cardiac and respiratory states must be centrally monitored in order to recognize and actively treat them [106].

Nitroglycerin is the first-line therapy for Irukandji syndrome-related hypertension. Its impact on venous and arterial dilatation provides advantages in victims with life-threatening pulmonary edema. In persistent hypertension, an infusion of nitroglycerin can be initiated and adjusted to the targeted blood pressure. Its usage is contraindicated in people using phosphodiesterase inhibitors, as it is in other uses of nitrates [33].

Phentolamine: Because of its alpha-adrenergic antagonist properties, phentolamine is also suggested as a therapy for Irukandji syndrome-related hypertension [119,164]. Because of the risk of hypotension, delayed cardiac failure, and pulmonary edema in extreme instances, phentolamine is preferred over phenoxybenzamine, due to it being reversible and having a shorter half-life. Nonetheless, unless contraindicated, a titratable vasodilator such as nitroglycerin should be administered initially, particularly in victims with concomitant heart failure, and phentolamine should be reserved for individuals resistant to nitrates [155].

Benzodiazepines: In Irukandji syndrome, benzodiazepines are advised as adjunctive therapy for pain and hypertension. Usually, a combination of suitable analgesics and benzodiazepines will alleviate the hypertension caused by Irukandji syndrome [217].

Magnesium sulfate (MgSO_4_) administration is a current practice [218]. However, there are no strong data to support or advise against this use [167]. The only randomized controlled trial on this topic showed no difference between MgSO_4_ administration and placebo on Irukandji patients, though minimal doses of MgSO_4_ and large exclusion criteria were applied [219]. The current practice is based on the effectiveness documented in case series. The reported symptom recurrence after dosage reduction suggests a dose-response association. More randomized controlled trials are necessary to establish the function of MgSO_4_ as part of the treatment of this envenomation. In the meantime, it can be considered in severe cases. 

### 8.3. Seabather´s Eruption

This syndrome is usually mild and benign. Management often includes topical and systemic antihistamines, as well as strong topical steroids. In some cases, fever and extensive lesions may demand a short course of oral glucocorticoids. Swimmers affected by seabather´s eruption should remove their swimsuit prior to showering, as the fresh water may cause further discharge. The swimwear must be thoroughly washed with soap before reuse to eliminate any remaining larvae [76,220].

### 8.4. Corneal Lesions

Jellyfish venom is harmful to the eyes as well. Stings cause damage to the eyelid, conjunctiva, cornea, and anterior chamber [221]. Following a sting, 3% NaCl and 0.3% Norfloxacin eyedrops have been successful in the treatment of epithelial keratitis and corneal edema. The lesion healed within 2 weeks, with minimal scarring [222]. Treatment with nematocyst removal, topical antihistamines, topical steroids, cycloplegics, and topical antibiotics has also been described, with good results [178]. In case of a *Physalia* ocular sting, it is recommended to inactivate the toxin with heat, apply analgesia, and debride the cornea [181].

### 8.5. Other Treatments

Systemic antihistamines, topical/oral corticosteroids, or tacrolimus ointment may be beneficial in severe delayed reactions [85,90,177]. Extracorporeal shock wave therapy was successful in the treatment of a chronic recurrent dermatitis following a *Physalia physalis* sting that was resistant to local cortisone treatment [93]. Early radical debridement and vacuum-assisted wound care appear to be useful in preventing skin necrosis and consequently progressive tissue loss caused by stings [82].

## 9. Forensic and Toxicological Aspects

Forensic identification of humans can be based on primary and secondary methods. The latter include anthropology, evidence, and medical and personal characteristics, such as scars and tattoos. Although categorized as secondary, these methods may play a fundamental role in the identification of a victim [223]. In some cases, jellyfish stings leave a permanent scar on the victim, becoming a distinctive characteristic of that person. As such, these stings can be used as a secondary identification method, improving identification accuracy.

Reports of postmortem examinations after a jellyfish sting are scarce, and mostly reveal cerebral hemorrhage, as well as visceral and cerebral congestion.

A 4-year-11-months-old boy was stung by a jellyfish and died 40 min later. His autopsy, 19 h hours after the death, revealed whitish foam in the oral endotracheal tube, a reticular pattern of eruptions on the left arm and left lateral thorax. The skin scraping of the lesions showed nematocyst similar to those of *Chiropsalmus quadrumanus*. A total of 75 mL of serous fluid was removed from each pleural space. The larynx, trachea, and bronchial tree were filled with frothy white foam. The lungs weighed 530 g and had a purple, congested appearance. The cut surface was edematous and congested, with a prominence of the septa. There was subendocardial hemorrhage on the septal wall of the left ventricle. Acute passive congestion was evident in the liver, spleen, and kidneys. Histological exam revealed widespread interstitial and perivascular lymphoid infiltration of the myocardium. There were a few areas of early changes of hypoxia, but no necrosis. There was focal subendocardial hemorrhage in the septum of the left ventricle. Many alveolar capillaries were congested with monocytes. Interstitial and perivascular lymphocytic infiltrates were also seen in the salivary glands, thyroid, trachea, esophagus, hepatic portal areas, and the dermis. Lymphoid nodules were found in the bone marrow [194].

A 67-year-old obese woman stung by a *Physalia physalis* became comatose shortly after and died 5 days later. Linear, erythematous, vesicular lesions were seen on her right arm, with a total length of about 75 to 350 cm. A microscopic inspection of the affected skin revealed numerous nematocysts. Subepidermal separations were apparent at the dermo-epidermal junction. Patchy necrosis with neutrophils and lymphocytes was detected in the epidermis, while erythrocyte extravasation was found in the dermis. There were focal regions of arteriosclerotic cardiovascular disease and signs of coagulopathy, but no myocardial death [224]. 

Postmortem examination of a previously healthy 44-year-old male diagnosed with Irukandji syndrome revealed a substantial intracranial hemorrhage with no arteriovenous malformations. The nematocyst retrieved from the skin of the patient matched those of *Carukia barnesi* [149].

## 10. Climate Changes and the Increased Risk of Jellyfish Stings

The rising water temperatures are expected to prompt jellyfish blooms, both by directly increasing asexual reproduction and by promoting eutrophization, therefore boosting jellyfish food availability. Specifically, the complex life cycle of scyphozoan jellyfish alternates between benthic (polyp) and pelagic (medusa) phases. Medusae reproduce sexually by producing planula larvae, which land on surfaces and evolve into polyps that reproduce asexually. Under specific environmental circumstances, a mechanism known as strobilation allows polyps to discharge microscopic jellyfish (ephyrae) into the water. Polyp asexual reproduction rate has been shown to increase with water temperature, up to a certain optimal point [225,226,227,228]. However, overwintering temperatures are required to enable strobilation, with some species tolerating higher strobilation temperatures, while others were unable to thrive in warmer conditions. As such, although it has been hypothesized that jellyfish will flourish in rising water temperatures, future climate change might prohibit species that can only strobilate under certain circumstances, limiting the biodivesrsity of jellyfish [229]. Eutrophication has also been implicated in jellyfish blooms. In fact, food availability greatly increases jellyfish populations, as it improves fertility and triggers a shift from asexual to sexual reproduction, resulting in more individuals in the medusa stage [230].

## 11. Conclusions

**Figure 11:**
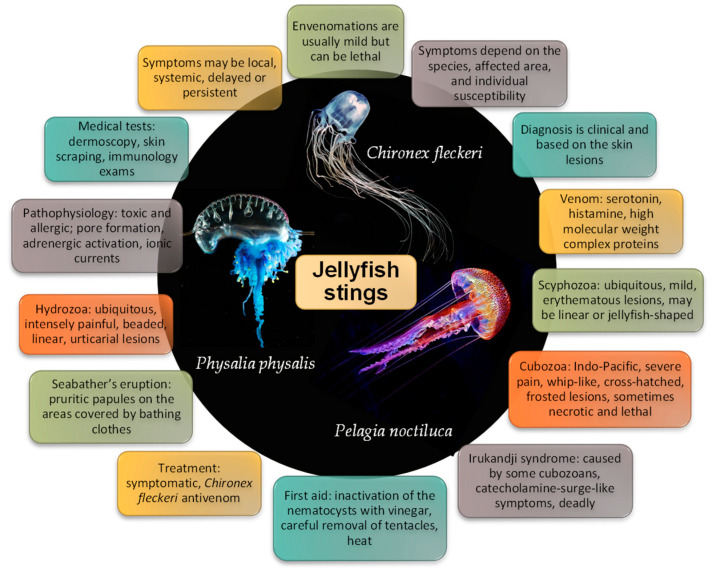
Major characteristics of jellyfish stings.

Major characteristics of jellyfish stings are summarized in Figure 11. Major characteristics of jellyfish stings. Most patients and doctors alike are unaware of the clinical picture and treatment of a jellyfish envenomation. Some stings, particularly those caused by scyphozoans (i.e., true jellyfish) are usually mild and limited to an erythematous rash, while others (mostly cubozoan stings) can be lethal. Seabather’s eruption is a peculiar syndrome caused by the larval form of some jellyfish (mostly *Linuche unguiculata*), consisting of a highly pruritic papular rash in the regions covered by bathing clothes. Irukandji syndrome also deserves particular attention, as it is severe and often lethal. It resembles a catecholamine surge, and almost all cases described occurred in the Indo-Pacific region. The diagnosis is mainly clinical, based on the history of sea bath, the characteristics of the skin lesions, and, possibly, systemic symptoms. Skin scraping/sticky tape, dermoscopy, and reflectance confocal microscopy may help determine the occurrence of a jellyfish sting and identify the responsible species. Immunological tests may aid in determining if a patient has been previously exposed to a jellyfish and the risk of developing an allergic reaction.

The treatment of jellyfish stings remains controversial. It is widely accepted that inactivating the nematocysts and preventing further venom discharge is of utmost importance. However, research is conflicting regarding the safest method to achieve that goal. Vinegar irrigation is the most consensual treatment for inactivating nematocysts and heat application has been reported to inactivate the venom and offer pain relief. Careful removal of the tentacles with tweezers is also advocated. Further research is needed in this regard, particularly randomized controlled trials in different places of the world, as different jellyfish species seem to react differently to the treatments. The only specific treatment for a cnidarian envenomation in use thus far is a *Chironex fleckeri* antivenom. Other antivenoms are still being developed but were not tested in humans [231,232]. Finally, the way in which the venom exerts its toxic effects on humans is yet to be fully clarified and that may help to develop targeted treatments for jellyfish stings.

## Figures and Tables

**Table 1:** Jellyfish envenomation symptoms.

Type of Reaction	Reaction	Class Implied	References
Local reactions	Vesiculourticarial, painful dermatitis	Scyphozoa, Hydrozoa, Cubozoa	[14,76]
	Scarring	Scyphozoa, Cubozoa	[77,78]
	Infection	Scyphozoa, Cubozoa	[79,80]
	Pruritus	Scyphozoa	[81]
	Delayed dermatitis	Hydrozoa, Scyphozoa, Cubozoa	[82,83,84,85,86,87,88,89]
	Recurrent dermatitis	Scyphozoa, Hydrozoa	[90,91,92,93]
Parasympathetic actions	Parasympathetic dysautonomia	Cubozoa	[94]
	Ileus paraliticus	unknown	[95]
	Urinary incontinence	Cubozoa	[94,96]
	Biliary dyskinesia	Scyphozoa	[96]
	Vascular spasm	Hydrozoa	[97,98,99]
Sympathetic actions	Mydriasis	Scyphozoa	[100]
	Blurred vision	Scyphozoa	[101]
	Stress cardiomyopathy	Cubozoa, Scyphozoa	[30,72]
	Irukandji syndrome	Cubozoa	[102,103,104,105,106,107,108]
Neuromuscular actions	Mononeuritis multiplex	unknown	[109,110]
	Peripheral neuropathy	Scyphozoa, Cubozoa	[99,111,112,113,114]
	Guillain-Barré	Scyphozoa	[115,116]
	Complex Regional Pain Syndrome	unknown	[117]
	Dysphonia	Hydrozoa, Scyphozoa	[13,118,119]
Other systemic reactions	Acute renal failure	Hydrozoa	[120]
	Arrythmia	Hydrozoa	[121]
	Rhabdomyolysis	Hydrozoa	[122,123]
	Erythema nodosum	Hydrozoa	[124]
	Deep venous thrombosis	Scyphozoa	[125]
	Facial swelling	unknown	[126,127]
	Digital necrosis	unknown	[128,129,130]
	Spinal cord infarct	unknown	[131]
	Cellulitis	Scyphozoa	[79]
	Myalgia	Scyphozoa, Cubozoa	[132]
	Abdominal cramping	Scyphozoa, Cubozoa	[13,119]
	Cough	Scyphozoa	[13,119]
	Nausea and vomiting	Hydrozoa	[133]
	Cold sweating	Hydrozoa	[133]
	Delayed eye swelling	unknown	[134]
Long term reactions	Keloids	Cubozoa	[71,73]
	Lichen planus	unknown	[135]
	Hyperpigmentation	Scyphozoa, Hydrozoa, Cubozoa	[78,86,136]
	Fat atrophy	unknown	[137]
	Delayed necrosis	Cubozoa	[82]
	Gangrene	Cubozoa	[138]
	Persistent hypersensitivity	Scyphozoa	[139]
	Koebner Phenomenon	unknown	[140]
	Persistent neuropathy	unknown	[111]
	Granulomatous infiltration	Cubozoa	[87]
	Nodular papular eruption	Cubozoa	[84]
Potentially fatal reactions	Immediate cardiac arrest	Cubozoa	[141,142,143]
	Acute myocardial infarction	unknown	[29,31]
	Rapid respiratory arrest	Cubozoa, Scyphozoa	[75,141]
	Anaphylaxis	Scyphozoa, Hydrozoa, Cubozoa	[121,144,145]
	Angioedema	Scyphozoa	[121,146]
	Pulmonary edema	Scyphozoa, Cubozoa	[75,146,147]
	Shock	Scyphozoa, Hydrozoa	[132]
	Intracerebral hemorrhage after severe hypertension	Cubozoa	[148,149]
